# P-259. Facilitators and Barriers for the Use of Ultraviolet-C Disinfection at VA Hospitals: A Qualitative Analysis

**DOI:** 10.1093/ofid/ofae631.463

**Published:** 2025-01-29

**Authors:** Kimberly C Dukes, Stacey Hockett-Sherlock, Cassie Cunningham Goedken, A M Racila, Julia Walhof, Daniel Suh, Michihiko Goto, Eli N Perencevich

**Affiliations:** University of Iowa Carver College of Medicine, Iowa City, Iowa; University of Iowa, Iowa City, Iowa; Iowa City VA Medical Center, Iowa City, Iowa; U Iowa Carver College of Medicine, Iowa City, Iowa; Iowa City VA Health Care System, Iowa City, Iowa; Iowa City VA Health Care System, Iowa City, Iowa; University of Iowa/Iowa City VAMC, Iowa City, Iowa; University of Iowa/Iowa City VAMC, Iowa City, Iowa

## Abstract

**Background:**

Ultraviolet-C (UV-C) disinfection has been increasingly used to enhance terminal room cleaning and prevent hospital pathogen transmissions. We reported that UV-C implementation in Veterans Affairs (VA) hospitals was associated with a 17% reduction in Gram-negative bloodstream infections (BSI), but with a significant facility-level variation. To investigate this variation, we evaluated facilitators and barriers to implementing UV-C disinfection at VA hospitals.

**Methods:**

We interviewed environmental management services (EMS) leaders, housekeepers, infection preventionists, and multidrug-resistant-organism prevention coordinators at 22 US VA hospitals that used UV-C during the study period. Interviews focused on the context of external and internal environments, organization, tools and technology, tasks, and persons. We conducted a thematic analysis on transcripts.

**Results:**

21 of 22 participating VA hospitals report using UV-C as an adjunct to 2-step manual room cleaning. 34 staff completed interviews. The frequency of UV-C disinfection, locations, and rationales for use varied across hospitals. Interviewees reported using UV-C in operating rooms and patient rooms most often. Room use varied (e.g., routine discharge, contact precautions, other reasons).

Barriers at some hospitals included leaders’ distrust of manufacturer claims for exposure times, risks, and efficacy; concerns that non-VA data may not apply to the VA context; and a perceived lack of UV-C’s impact on local infection rates. Other barriers include high patient loads requiring fast room turnovers, reduced staffing, equipment and maintenance costs, and competing priorities. Facilitators for UV-C use included COVID-19-related financial resources, collaborative communication between EMS, Nursing, and Infection Prevention, and professional competence and commitment to infection prevention.

**Conclusion:**

Given the reported barriers and facilitators, a UV-C implementation toolkit informed by VA staff feedback could improve acceptability of UV-C technology for staff and is worth further study. Interprofessional collaboration and leadership support could improve integration of UV-C into environmental cleaning best practices in VA hospitals.

**Disclosures:**

**Michihiko Goto, MD MSCI**, Merck: Grant/Research Support

